# Enhanced thermoregulation abilities of shortfin mako sharks as the key adaptive significance of regional endothermy in fishes

**DOI:** 10.1111/1365-2656.70116

**Published:** 2025-08-29

**Authors:** Soma Tokunaga, Wei‐Chuan Chiang, Itsumi Nakamura, Rui Matsumoto, Yuuki Y. Watanabe

**Affiliations:** ^1^ Department of Evolutionary Studies of Biosystems The Graduate University for Advanced Studies, SOKENDAI Hayama Kanagawa Japan; ^2^ Eastern Fishery Research Center Fisheries Research Institute, Ministry of Agriculture Chenggong Taiwan; ^3^ Institute for East China Sea Research, Organization for Marine Science and Technology Nagasaki University Nagasaki Japan; ^4^ Okinawa Churashima Foundation Research Institute Motobu Okinawa Japan; ^5^ Research Center for Integrative Evolutionary Science The Graduate University for Advanced Studies, SOKENDAI Hayama Kanagawa Japan

**Keywords:** biologging, body temperature, endothermy, marine predator, pelagic ocean

## Abstract

1. Some large, wide‐ranging teleosts and elasmobranchs are converged to have regional endothermy, retaining metabolic heat via vascular countercurrent heat exchangers. Yet, their adaptive significance remains debated. While previous studies proposed potential benefits of elevated body temperature, enhanced controllability of body temperature enabled by heat exchangers may also be important. Some endothermic teleosts (e.g. bigeye tuna) alter rates of body temperature change depending on dive phases to maximize foraging time in deep, cold waters while minimizing recovery time in shallow, warm waters. However, whether endothermic elasmobranchs possess similar abilities remains unclear.
2. Using animal‐borne tags, we recorded diving behaviours and muscle temperatures of shortfin mako sharks, a possible elasmobranch equivalent to bigeye tuna. Warming and cooling rates were estimated with a heat exchange model. Further, we conducted literature‐based, phylogenetically informed comparative analyses of heat exchange rates across 25 fish species (mass range, 0.01–1600 kg).
3. All four mako sharks repeatedly dived below the thermocline with ambient temperature changes of up to 7–14°C. On average, muscle temperatures were 1.5–3.9°C warmer than the ambient water. Two individuals dived deep (up to 286–327 m) and showed a 14–47 times higher warming rate than cooling rate, whereas the other two individuals that dived shallowly exhibited one to two times differences. One individual warmed its muscle above sea surface temperature before a deep dive, possibly preparing for the coming deep excursion using internal heat sources.
4. Comparative analyses showed that the ratio of warming to cooling rate and its range across individuals was larger in endothermic bigeye tuna, swordfish and mako sharks than in most other fishes.
5. Our results demonstrate that enhanced temperature controllability has convergently evolved among some endothermic teleosts and elasmobranchs that inhabit low‐to‐middle latitude waters with strong thermal gradients. By contrast, some other endothermic species (e.g. salmon sharks and Atlantic bluefin tuna) that migrate to subpolar waters are specialized for body temperature elevation. We propose that the controllability and elevation of body temperature have different adaptive significance, reflecting species' habitats and foraging ecology. Our findings help explain the diversity and success of endothermic fishes as apex predators across the world's pelagic oceans.

## Graphical abstract

We show that regionally endothermic shortfin mako sharks have enhanced thermoregulatory ability with an order higher warming rate than cooling rate during repeated dives, likely optimizing foraging efficiency. This ability, only comparable to bigeye tuna and swordfish in teleosts, represents the key adaptive significance of regional endothermy in fishes.

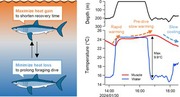

## 1 INTRODUCTION

The pelagic ocean is the largest biome on this planet, containing an estimated ~10 billion tons of fish biomass within its mesopelagic zones (200–1000 m) (Irigoien et al., 2014). Many large predatory fishes in the pelagic ocean, including tunas, billfishes and lamnid sharks, share a peculiar physiological characteristic, regional endothermy. Although most other fishes rapidly lose metabolic heat to the environment mainly through the gills (i.e. ectothermy) (Sorenson & Fromm, 1976), these fishes conserve metabolic heat through vascular countercurrent heat exchangers and maintain certain body parts warmer than the ambient water (Carey et al., 1971; Carey & Teal, 1966, 1969a). The warm parts range from slow‐twitch, aerobic red muscle (RM) (Carey & Teal, 1966, 1969a), internal organs (Carey et al., 1981; Carey & Lawson, 1973) and the eyes and brain (Block & Carey, 1985; Stevens & Fry, 1971). This unique thermal adaptation is a striking example of convergent evolution, because teleosts and elasmobranchs diverged as long as 450 million years ago (Ravi & Venkatesh, 2008).

Two primary hypotheses have been proposed about the potential adaptive significance driving the evolution of RM endothermy, the most common form of regional endothermy. The thermal niche expansion hypothesis states that RM endothermy provides some degree of thermal independence from the environments, enabling fish to expand their habitats across broad temperature ranges (Block, 1991; Carey et al., 1971; Carey & Lawson, 1973). The elevated cruising speed hypothesis argues that elevated RM temperatures enhance RM power output, thereby increasing cruising speeds and extending migration ranges (Carey & Teal, 1969a; Watanabe et al., 2015). While both hypotheses are still actively debated (Harding et al., 2021), they converge on the assumption that thermal insulation via heat exchangers, which leads to elevated RM temperature, primarily affects species' ecology and fitness. Curiously, however, the degree of temperature elevation varies greatly among species, ranging from a few degrees in the species inhabiting tropical and subtropical waters (e.g. yellowfin tuna *Thunnus albacares*) (Barrett & Hester, 1964) to 10–20 degrees in the species that migrate to subpolar waters (e.g. salmon sharks *Lamna ditropis*) (Goldman et al., 2004). This interspecific variation suggests that the adaptive significance of RM endothermy may not be attributed to RM temperature elevation alone.

The presence of heat exchangers may also enhance controllability of body temperature via active changes in heat exchange rate between body and the environment. This ability may be more important than body temperature elevation, especially in the species inhabiting low‐to‐middle latitude waters with strong thermal stratification. During deep foraging dives into mesopelagic zones, pelagic fishes often experience rapid decreases in the ambient temperature (Braun et al., 2019, 2022; Royer et al., 2023), potentially impairing their physiological performance and thereby limiting foraging time (Carey & Scharold, 1990; Nakamura et al., 2015). Yet, some endothermic teleosts, including bigeye tuna *Thunnus obesus* and swordfish *Xiphias gladius*, appear to overcome this challenge through the ability to switch their RM heat exchangers on and off (i.e. directing blood flow through or around them) depending on dive phases. This control leads to large changes in heat exchange rate, allowing them to decrease heat loss in deep, cold waters and increase heat gain in shallow, warm waters (Carey, 1990; Holland et al., 1992; Holland & Sibert, 1994; Malte et al., 2007; Stoehr et al., 2018). This ability should enhance foraging efficiency and ultimately fitness, potentially underpinning the evolution of RM endothermy. Crucially, however, previous studies on body temperature controllability predominantly examined endothermic teleosts, leaving it unknown whether endothermic elasmobranchs possess comparable abilities.

In this study, we used modern biologging technique (Watanabe & Papastamatiou, 2023) to record the diving behaviours and muscle temperatures of regionally endothermic shortfin mako sharks *Isurus oxyrinchus* (hereafter, mako sharks), a possible elasmobranch equivalent to bigeye tuna and swordfish. With their remarkable swimming ability (Waller et al., 2023), mako sharks regularly dive into prey‐rich mesopelagic zones, during which they experience rapid changes in ambient temperature (Braun et al., 2023; Vaudo et al., 2016). Although one study demonstrated their thermoregulatory abilities under captive conditions, the study subjects were limited to small juveniles (5.0–13.6 kg) due to logistic constraints (Bernal, Sepulveda, & Graham, 2001). Given that endothermic capabilities develop during growth (Kitagawa et al., 2022), sub‐adult or adult mako sharks are ideal elasmobranch models to test the idea of converged, high body temperature controllability in endothermic teleosts and elasmobranchs. We also compiled published data on heat exchange rates for 25 fish species, including both endothermic and ectothermic species, to compare the thermoregulatory abilities of mako sharks with those of other fishes. Combining these analyses, we explored a previously overlooked aspect (i.e. body temperature controllability) regarding the adaptive significance of regional endothermy in fishes.

## 2 MATERIALS AND METHODS

### 2.1 Field experiments

Field experiments were conducted with approval from the Animal Care Committee of the Fisheries Research Institute, Taiwan. We recorded diving behaviours and muscle temperatures of four female mako sharks captured by longline off Taitung (23.21°N, 121.48°E), south‐eastern Taiwan, during June–July 2023 and January 2024. Sharks were brought onboard to be measured and instrumented, and then released into the ocean. Body mass was estimated based on a length–weight relationship (Kohler et al., 1996). Our biologging package consisted of an accelerometer (W380‐PD3GT or W2000‐PD3GT, Little Leonardo), video camera (DVLW400M130‐4W or ‐4R, Little Leonardo), depth‐temperature recorder (LAT1810, Lotek), float, time‐scheduled release mechanism (RT‐4, Little Leonardo), satellite transmitter (SPOT6, Wildlife Computers) and radio transmitter (MM160B, Advanced Telemetry Systems). A package weighed 324 g, equivalent to 0.5%–1.5% of the estimated body mass of the sharks (22.3–70.4 kg). The accelerometer recorded depth, ambient water temperature and swim speed at 1 s intervals and tri‐axial acceleration at 1/16 or 1/32 s intervals for the full deployment period (18–30 h). The video camera recorded images of 640 × 480 pixels at 30 frames per second for 3.7–5.0 h, but the data showed no apparent prey capture events and were not used in this study. The LAT1810 recorded depth, ambient water temperature, and muscle temperature (measured by a 15‐cm stalk temperature sensor; see below) at 1 s intervals for the full deployment period. For one individual (Mako 2), the accelerometer failed to record data because of technical errors; however, data from the other loggers (depth‐temperature recorder and video camera) were successfully retrieved.

The package was secured on the shark's back anterior to the first dorsal fin by a plastic cable tie, which was passed through a small hole penetrated into the skin horizontally. Another small hole was vertically pierced to insert the stalk temperature sensor of LAT1810 into the deep white muscle, approximately 8 cm deep (Figure S1) (Nakamura et al., 2020; Watanabe et al., 2021). The entire package automatically detached from the shark by the time‐scheduled release mechanism (Watanabe et al., 2004) after 18–30 h of deployment. Upon the detachment, the package popped up to the surface and was located using the satellite and VHF transmitter, then retrieved by a boat.

To extend our dataset for interspecific comparison (see below), we also tagged a male tiger shark captured by a setnet off Okinawa Island, Japan, in December 2021. The experimental protocol was the same as for mako sharks.

### 2.2 Tag data analyses

The first 6 h of recording periods were excluded from the analyses to remove the potential effects of capture (Iosilevskii et al., 2022). To characterize diving behaviours of mako sharks, thermocline depth, which separates the warm mixed layer from the deep cold water and restricts the vertical movement of many fishes (Bernal et al., 2010, 2017), was determined for each individual shark. First, the depth and ambient temperature data, recorded at 1 s intervals, were averaged over 1 min intervals. Second, the data were grouped into 1 m depth bins, and the mean temperature for each bin was calculated. Third, the mean temperature values were smoothed using a 20‐m moving average. Thermocline depth was then identified as the point of the steepest temperature gradient (Figure S2). Deep dives of the sharks were extracted as excursions into the depth below thermocline; however, very short dives lasting for ≤10 min were excluded from the analyses. The body size dependence of dive duration and depth was examined by linear mixed models with shark ID (Mako 1–4) as a random effect. The relationship was considered statistically significant when the 95% CI of the regression slope did not include zero. These analyses were conducted using the software R ver. 4.3.1 (R Core Team, 2023) with the package ‘dplyr’ (Wickham et al., 2023), ‘zoo’ (Zeileis & Grothendieck, 2005), ‘lmerTest’ (Kuznetsova et al., 2017) and the software Igor Pro (WaveMetrics) with the package Ethographer (Sakamoto et al., 2009).

A heat exchange model was used to estimate the whole‐body heat transfer coefficients of sharks (Brill et al., 1994; Holland & Sibert, 1994). The rate of body temperature change at time t can be expressed as

(1) $$\frac{dT_{b}\left(t\right)}{\mathrm{dt}}=k\left(T_{a}\left(t\right)-T_{b}\left(t\right)\right)+\dot{T_{m}},$$

where $T_{b}$ is muscle temperature (°C), $T_{a}$ is ambient temperature (°C), k is whole‐body heat transfer coefcient (°C min^−1^ °C^−1^) and $\dot{T_{m}}$ is internal heat production rate (°C min^−1^). $\dot{T_{m}}$ was assumed to be constant due to relatively constant cruising speeds throughout the recording periods (Figure S3). As k may vary between the warming ($T_{a}\geq T_{b}$) and cooling phases ($T_{a}<T_{b}$), both constant and variable models were tested:

(2) k=aconstant value,

(3) $$k=\left\{\begin{array}{c} k_{\text{warm}};T_{a}\left(t\right)\geq T_{b}\left(t\right)\\ k_{\text{cool}};T_{a}\left(t\right)<T_{b}\left(t\right) \end{array}\right.,$$

where $k_{\text{warm}}$ and $k_{\text{cool}}$ are k during the warming and cooling phases, respectively. The muscle and ambient temperature data were subsampled at 1 min intervals to apply this model. The software R with package ‘minpack.lm’ (Elzhov et al., 2023) was used to select a combination of $k_{\text{warm}}$, $k_{\text{cool}}$ and $\dot{T_{m}}$ that best fit the muscle temperature traces based on the least squares method. The initial value of the simulated muscle temperature was set at the measured value for each shark, and that of $k_{\text{warm}}$, $k_{\text{cool}}$ and $\dot{T_{m}}$ was all set at 0.01 with the lower and upper limits of 0 and 1, respectively. The best model for each shark was determined based on the mean absolute error (MAE) and Akaike information criteria (AIC) (Akaike, 1987).

### 2.3 Comparative analyses

Heat transfer coefficients of various fishes estimated in captive or field experiments were compiled. We accepted only studies that estimated heat transfer coefficients separately during the warming and cooling phases, or during the corresponding ascent and descent phases. Although the heat exchange model was previously applied to Pacific bluefin tuna *Thunnus orientalis* with RM endothermy, $k_{\text{warm}}$ could not be estimated because the body was consistently warmer than the ambient water (Kitagawa et al., 2001). When the individual data were presented graphically rather than numerically, the values were extracted from the figures using the software Graphcel (ver. 1.11; http://www.vector.co.jp/soft/dl/win95/business/se247204.html). The allometric relationships between heat transfer coefficients and body mass were examined using the software R with the package ‘MCMCglmm’ (Hadfield, 2010) to account for the phylogenetic non‐independence of data (Felsenstein, 1985) and intraspecific variations as random effects simultaneously. It was set to run for 500,000 iterations, with a sampling interval of 500 after removing the first 50,000 iterations as burn‐in. A phylogenetic tree was created using the software Mesquite (Maddison & Maddison, 2019) with the phylogenetic relationships provided in ‘Chondrichthyan Tree of Life’ (Naylor, 2024) for elasmobranchs and in ‘The Fish Tree of Life’ (Rabosky et al., 2018) for teleosts, with an arbitrary branch length (Grafen, 1989; Figure S4). Response variables included heat transfer coefficient (i.e. $k_{\text{warm}}$, $k_{\text{cool}}$ or $k_{\text{warm}}/k_{\text{cool}}$), while explanatory variables included body mass, whether the fish has regional endothermy, and their interaction. All continuous variables (i.e. $k_{\text{warm}}$, $k_{\text{cool}}$, and $k_{\text{warm}}/k_{\text{cool}}$ and body mass) were log_10_‐transformed to improve the linearity of relationships among the variables. The best model was determined based on the deviance information criterion (DIC) (Spiegelhalter et al., 2002).

## 3 RESULTS

**FIGURE 1:**
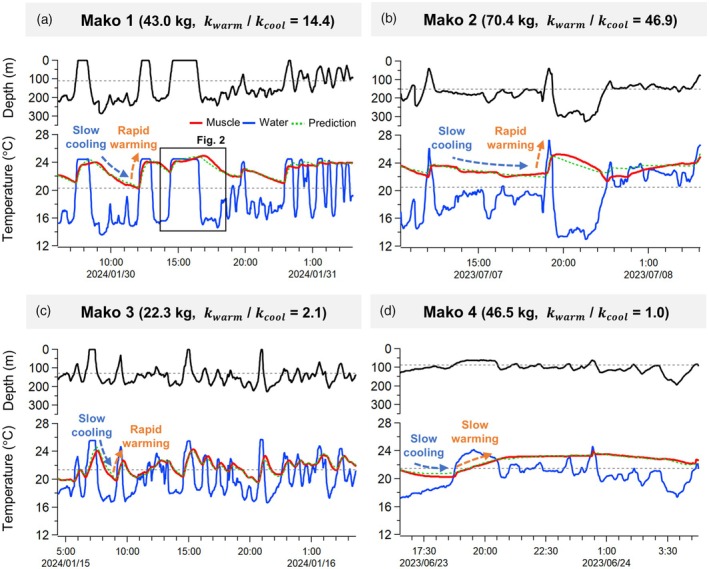
Diving behaviours and muscle temperatures of regionally endothermic shortfin mako sharks. Panels (a–d) correspond to individual shark (Mako 1–4), respectively. The muscle (red) and ambient temperature (blue), as well as the predicted muscle temperature trace from heat exchange model (green dotted) were shown with depth (black) for each individual. The grey dotted lines represent the thermocline depth and corresponding ambient temperature. Mako sharks altered their heat exchange rates during deep dives below the thermocline, except for Mako 4 with less vertical movement (d). The enclosed area in (a) was enlarged in Figure 2.

We recorded the diving behaviour, ambient temperature and muscle temperature (deep white muscle near RM; see Section 2) of four female mako sharks (Mako 1–4) ranging from 22.3 to 70.4 kg in estimated body mass (Figure 1; Table 1). They repeatedly dived below the thermocline of 87–150 m depth, reaching maximum depths of 227–327 m and experiencing large temperature changes of 7.4–14.3°C (range across individuals). The muscle temperatures exceeded the ambient temperatures for 74–88% of the time, with the average and maximum elevation of 1.5–3.9°C and 5.3–12.0°C, respectively. They returned to shallow warm waters above the thermocline before their muscle temperatures dropped below 20°C in most cases. The duration of deep dives (defined as periods spent below the thermocline for over 10 min) reached a maximum of 133–324 min and significantly increased with body mass (Figure S5). However, one middle‐sized individual (Mako 4) remained at relatively shallow depths just below the thermocline and exhibited fewer vertical movements compared to other individuals (Figure 1d). Consequently, there was no significant relationship between dive depth and body mass (Figure S5).

**TABLE 1:** Descriptive information and summary for depth and temperature data of tagged mako sharks.

Shark ID	Fork length (cm)	Body mass (kg)	Recording period (h)^a^	Depth (m)^b^	Ambient temperature (°C)^b^	Muscle temperature (°C)^b^	Temperature difference (°C)^c^	Dive duration (min)	Dive depth (m)
				Mean (range)	Mean (range)	Mean (range)	Mean (range)	Mean (range)	Mean (range)
Mako 1	159	43.0	28.0	135 (0–286)	19.2 (13.6–24.6)	23.0 (20.2–25.0)	3.8 (−2.4–10.0)	106 (18–222)	207 (150–286)
Mako 2	186	70.4	23.7	176 (39–327)	19.3 (13.0–27.3)	23.1 (21.4–25.3)	3.9 (−3.9–12.0)	124 (16–324)	235 (165–327)
Mako 3	129	22.3	30.3	143 (0–227)	20.2 (16.6–25.8)	21.7 (19.6–24.3)	1.5 (−5.1–6.3)	53 (17–133)	181 (144–227)
Mako 4	163	46.5	18.3	102 (60–194)	20.7 (17.2–24.6)	22.4 (20.3–23.9)	1.8 (−2.5–5.3)	71 (35–149)	127 (106–194)

^a^ The first 6 h of recording periods were excluded from the analyses to remove the effects of capture.

^b^ The data was subsampled at 1 min intervals.

^c^ The difference between the muscle temperature and ambient water temperature, with positive values indicating elevated muscle temperature.

To assess their capacity for physiological thermoregulation, we applied a heat exchange model to estimate the whole‐body heat transfer coefficient, k (the rate of body temperature change for a unit temperature difference between body and the ambient water) and internal heat production rate (Table 2; see Section 2). For Mako 1–3, the models with variable k between the warming ($k_{\text{warm}}$) and cooling phases ($k_{\text{cool}}$) showed lower fitting errors (mean absolute error, MAE) and AIC than those with a constant k. The large (Mako 2), medium (Mako 1) and small‐sized (Mako 3) sharks showed 46.9, 14.4 and 2.1 times higher $k_{\text{warm}}$ than $k_{\text{cool}}$, respectively. In contrast, the fitting errors of both models were similar for Mako 4 (0.164 and 0.157°C for variable and constant k model, respectively), indicating an overfitting of the variable k model. The constant k of this individual (0.0058) was closer to $k_{\text{cool}}$ (0.0016–0.015) than to $k_{\text{warm}}$ (0.032–0.075) of other individuals.

**TABLE 2:** Estimated parameters of the heat exchange model for each mako shark.

Shark ID	Model	k	$k_{\text{warm}}$	$k_{\text{cool}}$	$k_{\text{warm}}/k_{\text{cool}}$	$\dot{T_{m}}$	MAE	AIC	ΔAIC
Mako 1	(1)	0.0071	—	—	1.0	0.029	0.566	−901.4	2217.7
	**(2)**	—	**0.052**	**0.0036**	**14.4**	**0.0062**	**0.243**	**−3119.1**	**0**
Mako 2	(1)	0.0015	—	—	1.0	0.0068	0.652	−388.6	977.7
	**(2)**	**—**	**0.075**	**0.0016**	**46.9**	**0.0017**	**0.405**	**−1366.3**	**0**
Mako 3	(1)	0.019	—	—	1.0	0.030	0.291	−2969.6	760.3
	**(2)**	**—**	**0.032**	**0.015**	**2.1**	**0.016**	**0.216**	**−3729.9**	**0**
Mako 4	**(1)**	**0.0058**	—	—	**1.0**	**0.012**	**0.164**	**−2212.0**	**94.4**
	(2)	—	0.0021	0.0072	0.29	0.015	0.157	−2306.4	0

*Note*: Model (1) and (2) correspond to the constant and variable k model, respectively. The best models were denoted by bolds. Although the variable k model showed lower AIC than the constant k model for all individuals, the fitting errors (mean absolute error, MAE) for Mako 4 were similar for both models, indicating that the variable k model was overfitted.

**FIGURE 2:**
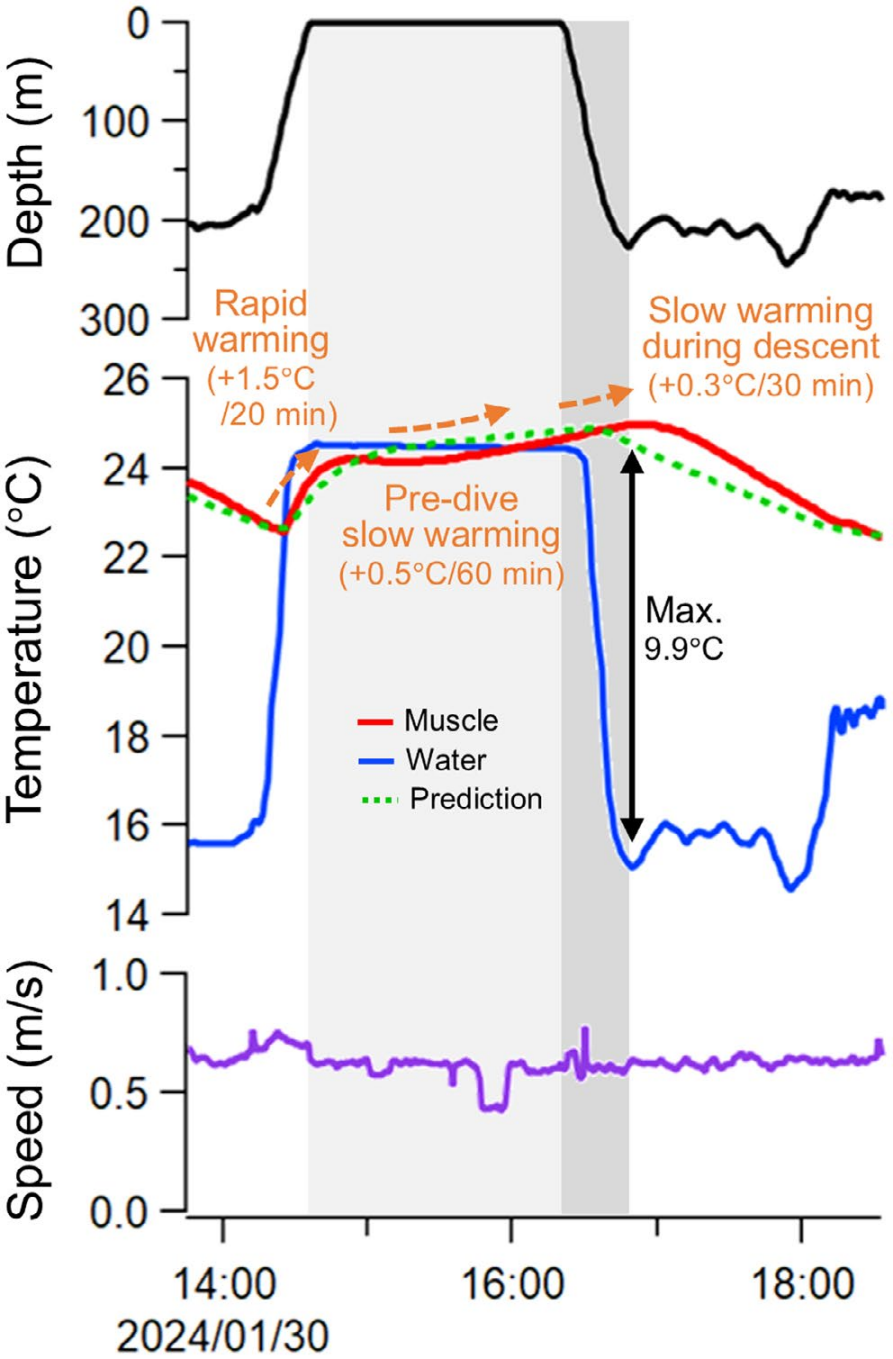
Muscle temperature trace of Mako 1 that could not be fully explained by the heat exchange model. Background colours represent the surface period (light grey) and the descent period (dark grey). This individual rapidly recovered its muscle temperature (red), and then further increased it above the ambient temperature (blue) at the surface prior to a deep dive. Muscle temperature continued to rise during the subsequent descent phase, and reached a peak at a cold depth. The model prediction (green dotted) failed to replicate these temperature changes. The depth and swim speed during this period, subsampled at 1 min intervals, were also shown.

The heat exchange model failed to replicate some parts of the muscle temperature trace of Mako 1 (Figure 2). Following a deep dive, this individual rapidly recovered its muscle temperature by 1.5°C within 20 min in shallow warm waters above the thermocline, then further increased it by 0.5°C over the 60 min at the surface prior to the next deep dive. Remarkably, muscle temperature exceeded the ambient temperature during the latter period. This result indicates that the shark used internal (i.e. metabolic) heat sources, although some effects of solar radiation cannot be excluded due to the shallow swimming depth (~0 m) and time of the day (from 3:20 to 4:20 PM local time) during the period. During the subsequent descent, muscle temperature continued to rise and reached a peak of 25.0°C at a depth of 211 m, resulting in a maximum body temperature elevation above the ambient water of 9.9°C, as opposed to the model prediction of decreasing muscle temperature during the descent phase.

**FIGURE 3:**
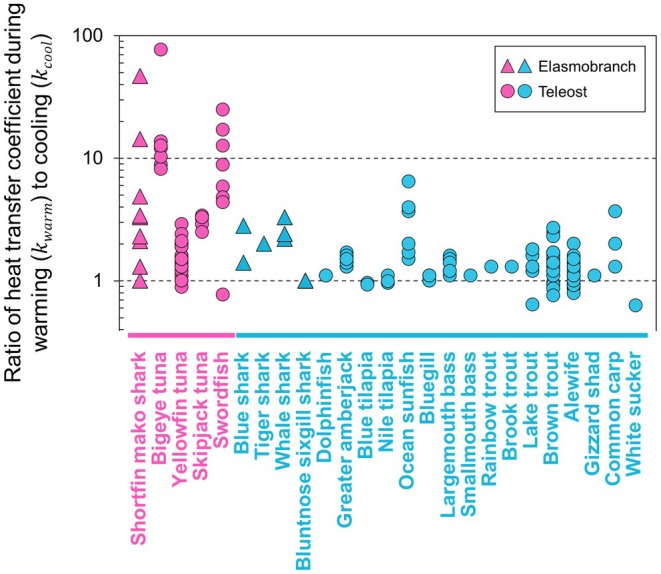
Ratio of whole‐body heat transfer coefficient during warming to cooling across 25 fish species. Values for individual fish of regionally endothermic (pink) and ectothermic species (sky‐blue) were plotted. Data from both field and captive studies were compiled. In shortfin mako sharks, values from captive experiment (*N* = 5) (Bernal, Sepulveda, & Graham, 2001) were added to our data from the field (*N* = 4). Endothermic mako sharks, bigeye tuna and swordfish exhibited an ability to alter their heat exchange rates by over tenfold, unlike ectothermic fishes. The interspecific variations were also much larger in these species. See Table S1 for the dataset used in this study.

**FIGURE 4:**
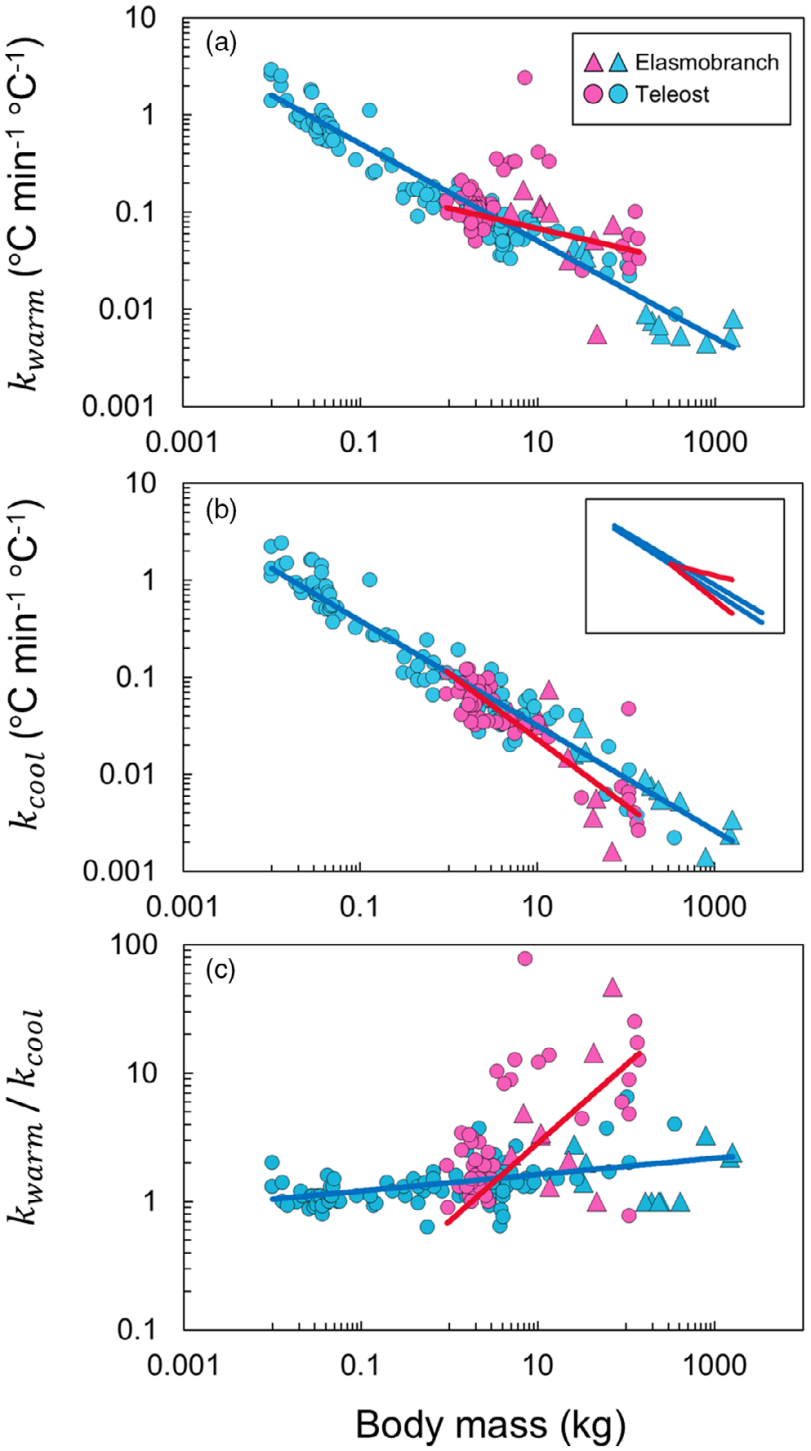
Allometric relationships between heat transfer coefficients and body mass in fishes. Heat transfer coefficients during warming (a) and cooling (b) and their ratio (c) were plotted against body mass for individual fish of 25 species. The regression equations are as follows: $k_{\text{warm}}$ = 0.11 × Mass^−0.21^, $k_{\text{cool}}$ = 0.11 × Mass^−0.68^, $k_{\text{warm}}/k_{\text{cool}}$ = 0.70 × Mass^0.61^ for endothermic fishes (red solid lines) and $k_{\text{warm}}$ = 0.16 × Mass^−0.50^, $k_{\text{cool}}$ = 0.11 × Mass^−0.54^, $k_{\text{warm}}/k_{\text{cool}}$ = 1.4 × Mass^0.064^ for ectothermic fishes (blue solid lines). Inset in (b) shows a comparison of four regression lines in (a) and (b). For each colour, the regression line from (a) is positioned above that from (b). See Table S1 for the dataset used in this study.

We compiled data on heat transfer coefficients during warming and cooling phases estimated in captive or field experiments for 25 fish species (teleosts and elasmobranchs), including five species with RM endothermy. Body mass ranged from 10 g to 1600 kg (Table S1). The ability to alter heat exchange rate, as assessed by $k_{\text{warm}}/k_{\text{cool}}$ for individual fish, was enhanced (over tenfold) in endothermic mako sharks, bigeye tuna and swordfish (Figure 3). Other fishes, including endothermic yellowfin tuna and skipjack tuna *Katsuwonus pelamis*, typically had the values of <4. Intraspecific variations across individuals in $k_{\text{warm}}/k_{\text{cool}}$ were also exceptionally large, spanning an order of magnitude, in mako sharks and swordfish. Phylogenetically informed regression analyses revealed that $k_{\text{warm}}$, $k_{\text{cool}}$ and their ratio change allometrically with body mass (Figure 4). The best models included body mass, whether the fish has RM endothermy, and their interaction as explanatory variables (Table 3). Due to the different slopes of regression models, the differences between endothermic and ectothermic fishes became more pronounced with increasing body mass. That is, large endothermic fishes exhibited higher $k_{\text{warm}}$ (Figure 4a), lower $k_{\text{cool}}$ (Figure 4b) and higher $k_{\text{warm}}/k_{\text{cool}}$ (Figure 4c) than similar‐sized ectothermic fishes. For the maximum body mass of endothermic fish in our dataset (140 kg), these differences were 2.9, 2.0 and 7.4 times, respectively.

**TABLE 3:** Results of the regression analyses on heat transfer coefficients as response variable.

Model	DIC	ΔDIC
**log**_**10**_($k_{\text{warm}}$) ~ **log**_**10**_(**Mass**) + **Endothermy** + **log**_**10**_ (**Mass**)*Endothermy	**−105.11**	**0**
log_10_($k_{\text{warm}}$) ~ log_10_(Mass) + Endothermy	−102.99	2.12
log_10_($k_{\text{warm}}$) ~ log_10_(Mass)	−102.87	2.24
**log**_**10**_($k_{\text{cool}}$) ~ **log**_**10**_(**Mass**) + **Endothermy** + **log**_**10**_(**Mass**)*Endothermy	**−78.81**	**0**
log_10_($k_{\text{cool}}$) ~ log_10_(Mass) + Endothermy	−75.89	2.92
log_10_($k_{\text{cool}}$) ~ log_10_(Mass)	−69.80	9.01
**log**_**10**_($k_{\text{warm}}/k_{\text{cool}}$) ~ **log**_**10**_(**Mass**) + **Endothermy** + **log**_**10**_(**Mass**)*Endothermy	**−47.24**	**0**
log_10_($k_{\text{warm}}/k_{\text{cool}}$) ~ log_10_(Mass) + Endothermy	−34.78	12.46
log_10_($k_{\text{warm}}/k_{\text{cool}}$) ~ log_10_(Mass)	−34.41	12.83

*Note*: The best models with lowest DIC were denoted by bolds. The regression lines are shown in Figure 4.

## 4 DISCUSSION

### 4.1 Enhanced thermoregulation abilities of mako sharks

We demonstrated that mako sharks have enhanced thermoregulatory ability with an order higher warming rate than cooling rate during repeated dives below the thermocline (Figure 1). This ratio is comparable to those of bigeye tuna and swordfish, and is much larger than typical values of <4 reported for other fish species studied to date. As in bigeye tuna and swordfish, this ability allows mako sharks to prolong excursions in deep, cold waters and reduce the time needed for body temperature recovery at the surface, thereby likely optimizing foraging efficiency. Indeed, mako sharks conducted deep dives for up to 2–5 h, which are longer than the repeated 1‐h dives of ectothermic blue sharks *Prionace glauca* tagged at the same study site (Watanabe et al., 2021). Unlike bigeye tuna and swordfish, however, mako sharks lack the anatomical bypass route to switch RM heat exchangers off to facilitate heat exchanges with the ambient water (Bernal, Dickson, et al., 2001; Bernal, Sepulveda, & Graham, 2001; Malte et al., 2007; Stoehr et al., 2018). A possible mechanism is that they can change the efficiency of heat exchangers through the controls of blood flow rate (Scholander & Schevill, 1955). Notably, larger individuals stayed below the thermocline for longer periods and had larger changes in heat exchange rates. Similarly, a previous study showed that larger mako sharks tended to dive deeper (Sepulveda et al., 2004). These findings suggest that ontogenetic development of thermoregulatory abilities enables longer deep dives in larger mako sharks, although our relatively small sample size (*N* = 4) precludes firm conclusion. Additionally, one individual (Mako 4) that exhibited fewer vertical movements maintained a constant, low heat exchange rate during both warming and cooling phases, resulting in stable muscle temperature. This finding suggests that mako sharks can deactivate the ability to modulate heat exchange rate when it is not necessary.

During a surface period between deep dives, one mako shark increased its muscle temperature above the ambient temperature, a pattern that cannot be fully explained by the simple heat exchange model (Figure 2). This phenomenon possibly represents a physiological preparation for the coming dive into deep cold waters. The surface period included two distinct phases: first, muscle temperature was rapidly recovered, presumably through heat absorption from warm surface waters. Second, muscle temperature gradually rose above the ambient temperature, which was likely achieved by reducing the heat exchange rate and using metabolic heat. These observations suggest an active shift from external to internal heat sources for muscle warming via the control of heat exchanger efficiency, although the potential role of solar radiation (i.e. basking) cannot be entirely ruled out given that the shark remained near the sea surface (~0 m depth) during this period (Figure 2). Supporting this notion, the slow muscle warming continued during the subsequent dive (discussed below), suggesting that the heat exchange rate was minimized and maintained throughout the pre‐dive and descent phases. This pre‐dive muscle warming is notable in the context of the cognitive abilities of fishes, because, to our knowledge, no other species has been reported to change physiological states according to the actions they will take in hours.

Furthermore, this individual continued to increase its muscle temperature even during the subsequent descent phase for approximately 30 min, reaching its peak at a cold depth of >200 m (Figure 2). Cruising speed was not elevated during the descent, suggesting that the muscle temperature elevation was not caused by increasing metabolic heat production while swimming. A possible explanation is that the blood warmed at the surface circulates throughout the body during the subsequent dive. Consistent with this scenario, time lags of up to 20 min between the initiations of changes in the ambient and muscle temperature were reported in small mako sharks in captivity (Bernal, Sepulveda, & Graham, 2001). Measuring heart rates in large, wild mako sharks would be fruitful in future studies to elucidate the physiological mechanisms underlying their extraordinary thermoregulation abilities.

### 4.2 Adaptive significance of RM endothermy in fishes

Our comparative analyses showed that the high controllability of heat exchange rate, represented by an order higher $k_{\text{warm}}$ than $k_{\text{cool}}$, has convergently evolved among endothermic teleosts and elasmobranchs (Figure 3). Mako sharks and swordfish showed large intraspecific variations in $k_{\text{warm}}/k_{\text{cool}}$, indicating that their flexibilities in controlling heat exchange rates depend on the situations or undergo ontogenetic changes. Given the unique, pre‐dive muscle warming discussed above, mako sharks would represent an extreme case of high body temperature controllability. This convergent ability should provide a key adaptive significance by mitigating thermal constraints in the pelagic environments with strong vertical thermal gradient. We also found that large endothermic fishes had a higher $k_{\text{warm}}$, lower $k_{\text{cool}}$, and thus higher $k_{\text{warm}}/k_{\text{cool}}$ than similar‐sized ectotherms, while small endothermic fishes showed similar values to ectotherms (Figure 4). One possible reason for this result is that the small endotherms in our dataset were yellowfin and skipjack tuna, which typically forage in the warm mixed layer above the thermocline (Bernal et al., 2017). They are presumably under weaker selective pressure for modulating heat exchange rate, compared to larger species including mako sharks, bigeye tuna, and swordfish that repeat deep foraging dives below the thermocline.

**FIGURE 5:**
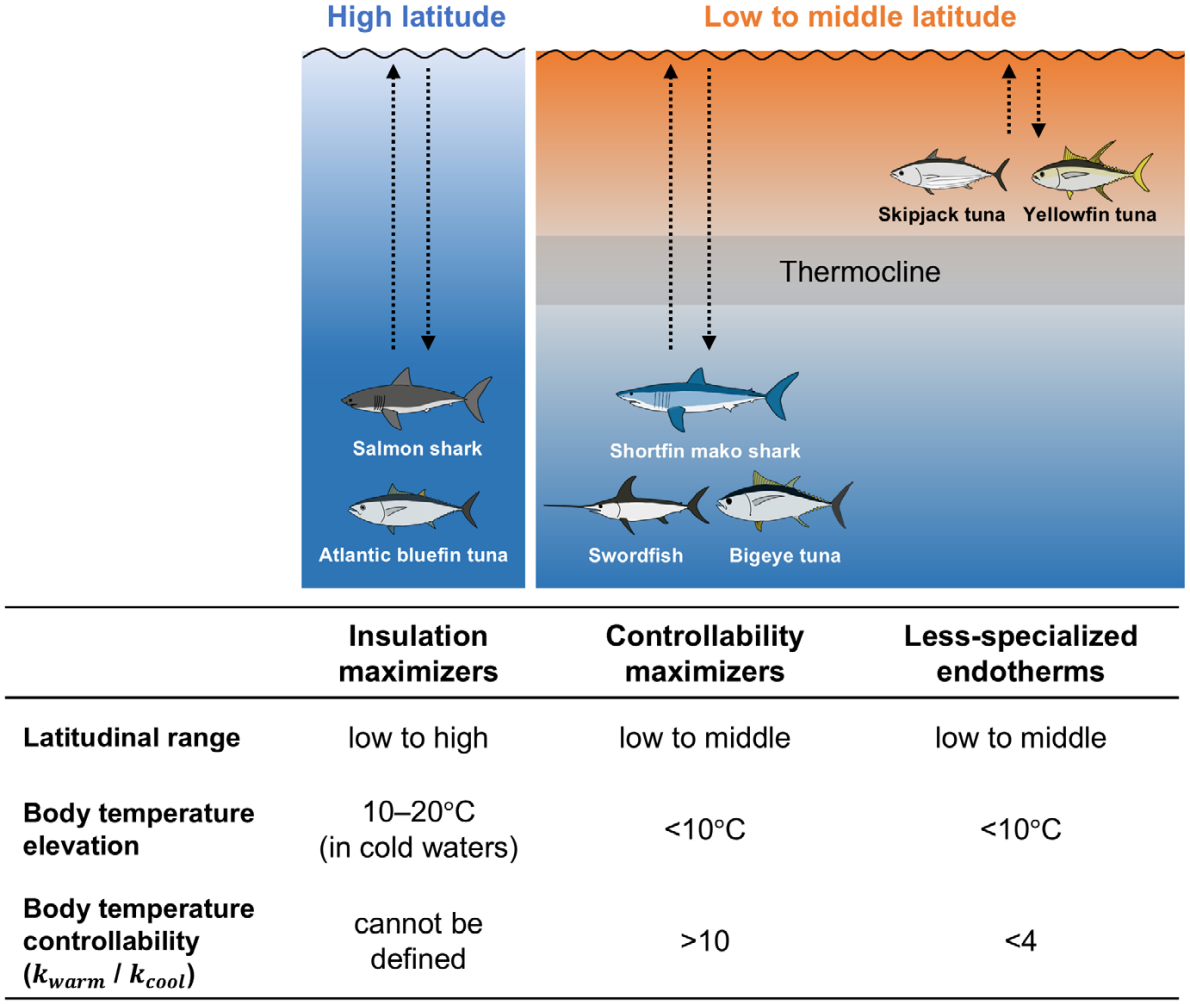
Schematic representations of spatial distribution and thermoregulatory ability in different types of regionally endothermic fishes. The dotted arrows represent vertical movements of fishes. There are two possible major evolutionary pathways, one enhancing body temperature elevation (i.e. ‘insulation maximizers’) and the other enhancing body temperature controllability (i.e. ‘controllability maximizers’). Some species have less‐specialized thermoregulatory abilities with preferences for shallow warm waters. Typical values for body temperature elevation above the ambient water temperature were based on previous measurements (Bernal et al., 2010).

Combining our findings with those of previous studies (Block et al., 2005; Carey & Teal, 1969b; Goldman et al., 2004; Teo et al., 2007; Weng et al., 2005), we propose that thermoregulatory abilities of teleosts and elasmobranchs with RM endothermy are converged in two different directions (Figure 5). One direction, stressed in previous studies, is the ability to maintain elevated body temperature by achieving thermal independence from the environments via heat exchangers. This ability is represented by what we term ‘insulation maximizers’, including salmon sharks and Atlantic bluefin tuna *Thunnus thynnus*, which can stay in subpolar waters for prolonged periods (Block et al., 2005; Weng et al., 2005). Salmon sharks maintain their body temperature constant in cold waters below 10°C, with the maximum temperature elevation of 21°C (Goldman et al., 2004). This body temperature stability could not be explained by the simple heat exchange model, which assumes heat transfer from warmer to cooler adjacent objects (Goldman et al., 2004). Thus, their body temperature controllability cannot be assessed by k values. Atlantic bluefin tuna have similar heat conservation ability, maintaining body temperatures up to 20°C above the ambient in cold waters (Carey & Teal, 1969b). Although they can alter their heat exchange rates over long periods (e.g. between day and night) (Teo et al., 2007), they seem unable to modulate them by more than tenfold depending on dive phases, unlike mako sharks and bigeye tuna. Closely related species such as porbeagle sharks *Lamna nasus* and Pacific bluefin tuna may also fall into this category, considering their high capacity for heat conservation and tolerance to cold waters (Carey & Teal, 1969a; Kitagawa et al., 2009).

The other direction is the ability to control heat exchange rate depending on the situation (especially dive phases), represented by what we term ‘controllability maximizers’, including mako sharks, bigeye tuna and swordfish, which inhabit tropical to warm‐temperate waters (Figure 5). Their body temperature elevation (typically <10°C) is not as pronounced as that of insulation maximizers. Instead, these species can change heat exchange rates by over tenfold, thereby increasing foraging efficiency during dive‐surface cycles in thermally stratified waters. Yellowfin and skipjack tuna could be regarded as less specialized forms of this type, given their preferences for shallow warm waters and less developed thermoregulatory capacity. Other endothermic elasmobranch species, such as white sharks *Carcharodon carcharias* with similar latitudinal distribution and diving capability to mako sharks (Block et al., 2011; Weng et al., 2007), could also belong to this type, although information is currently limited. In addition, some lamniform species thought to be ectotherms (i.e. basking sharks *Cetorhinus maximus* and smalltooth sand tiger *Odontaspis ferox*) were recently shown to have anatomical features suggestive of regional endothermy (Dolton, Jackson, et al., 2023; Dolton, Snelling, et al., 2023; Klöcker et al., 2025). Further research is needed to understand species‐specific thermoregulation abilities in endothermic elasmobranchs. Overall, thermoregulatory abilities of endothermic fishes could be grouped into two types that reflect species' latitudinal ranges and foraging depths. Our proposal provides a new perspective for understanding the adaptive significance and evolutionary drivers of fishes with RM endothermy, which dominate pelagic oceans as apex predators on a global scale.

In conclusion, we demonstrate that mako sharks possess enhanced thermoregulation abilities with an order higher warming rate than cooling rate, allowing them to flexibly control heat gain and loss according to dive phases. These abilities help mitigate the thermal constraints and optimize foraging efficiency in the low‐to‐middle latitude pelagic ocean. Moreover, our comparative analyses revealed the convergence of thermoregulatory abilities between endothermic elasmobranchs and teleosts. Our findings indicate that, in addition to body temperature elevation, high controllability of heat exchange rate is a key adaptive significance of regional endothermy in fishes, underpinning their remarkable success across the world's pelagic oceans.

## AUTHOR CONTRIBUTIONS

Soma Tokunaga, Wei‐Chuan Chiang and Yuuki Y. Watanabe conceived the ideas and designed methodology; Soma Tokunaga, Wei‐Chuan Chiang, Itsumi Nakamura, Rui Matsumoto and Yuuki Y. Watanabe collected the data; Soma Tokunaga analysed the data; Soma Tokunaga and Yuuki Y. Watanabe led the writing of the manuscript. All authors contributed critically to the drafts and gave final approval for publication.

## CONFLICT OF INTEREST STATEMENT

The authors declare no conflict of interest.

## Supporting information


**Figure S1.** Transverse section anterior to the first dorsal fin of a mako shark (180 cm in FL), obtained at the Shinkang fish market, Taiwan.
**Figure S2.** Depth‐temperature profiles for Mako 1–4 (A–D).
**Figure S3.** Swim speed of tagged mako sharks.
**Figure S4.** Phylogenetic tree used in comparative analyses.
**Figure S5.** Body mass dependence of dive duration (A) and dive depth (B) in four tagged mako sharks.
**Table S1.** Summary of body mass and heat transfer coefficients for 25 fish species.

## Data Availability

Data available in Figshare: https://doi.org/10.6084/m9.figshare.28202000.v1 (Tokunaga et al., 2025).
