# Supplementary material for: Enhanced thermoregulation abilities of shortfin mako sharks as the key adaptive significance of regional endothermy in fishes
Source: J Anim Ecol. 2025 Aug 29;94(11):2178–89. doi: 10.1111/1365-2656.70116 (PMC12586780; doi:10.1111/1365-2656.70116)
Supplement: Supplementary file 1 — Figure S1. Transverse section anterior to the first dorsal fin of a mako shark (180 cm in FL), obtained at the Shinkang fish market, Taiwan. Figure S2. Depth‐temperature profiles for Mako 1–4 (A–D). Figure S3. Swim speed of tagged mako sharks. Figure S4. Phylogenetic tree used in comparative analyses. Figure S5. Body mass dependence of dive duration (A) and dive depth (B) in four tagged mako sharks. Table S1. Summary of body mass and heat transfer coefficients for 25 fish species. [file JANE-94-2178-s001.pdf]

Supporting Information

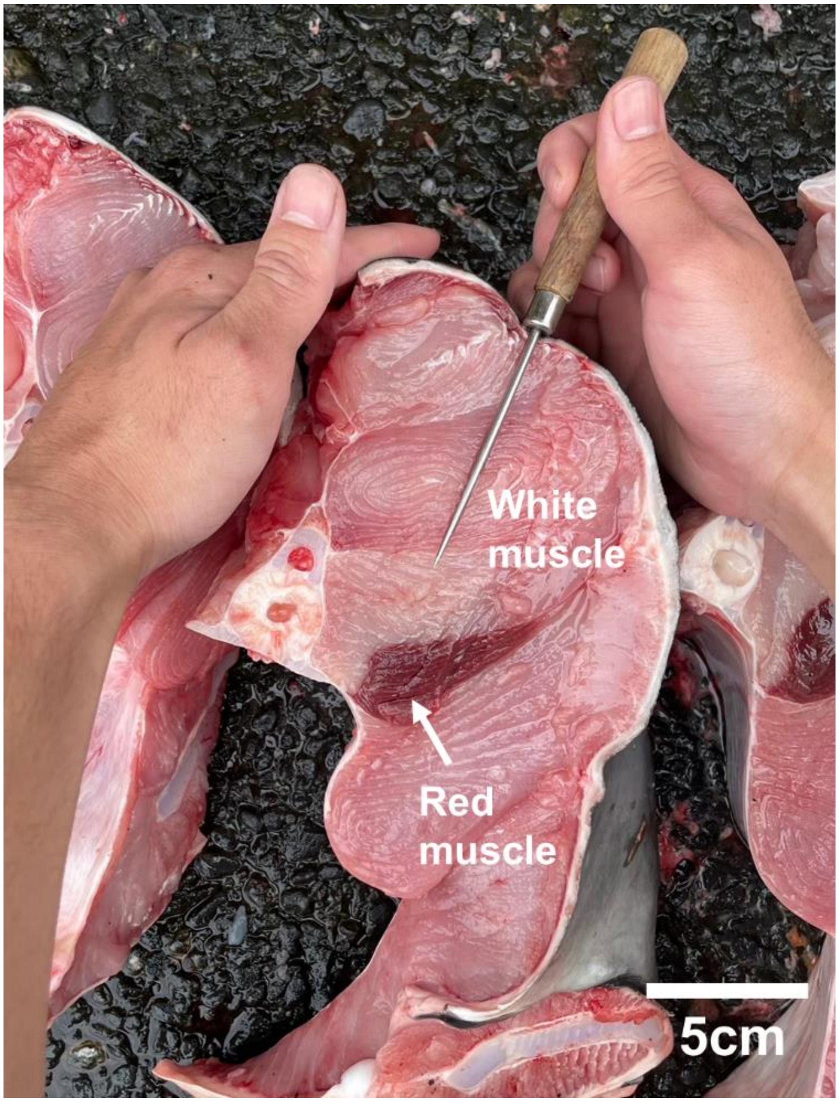

Figure S1. Transverse section anterior to the first dorsal fin of a mako shark (180 cm in FL), obtained at the Shinkang fish market, Taiwan. The tip of the needle located 8 cm deep from the body surface indicates the position reached by the stalk temperature sensor. Assuming the proportional growth of body depth, the temperature sensor reached the deep white muscle located approximately 1.8–2.6 cm away from the red muscle in tagged sharks (129–186 cm in FL).

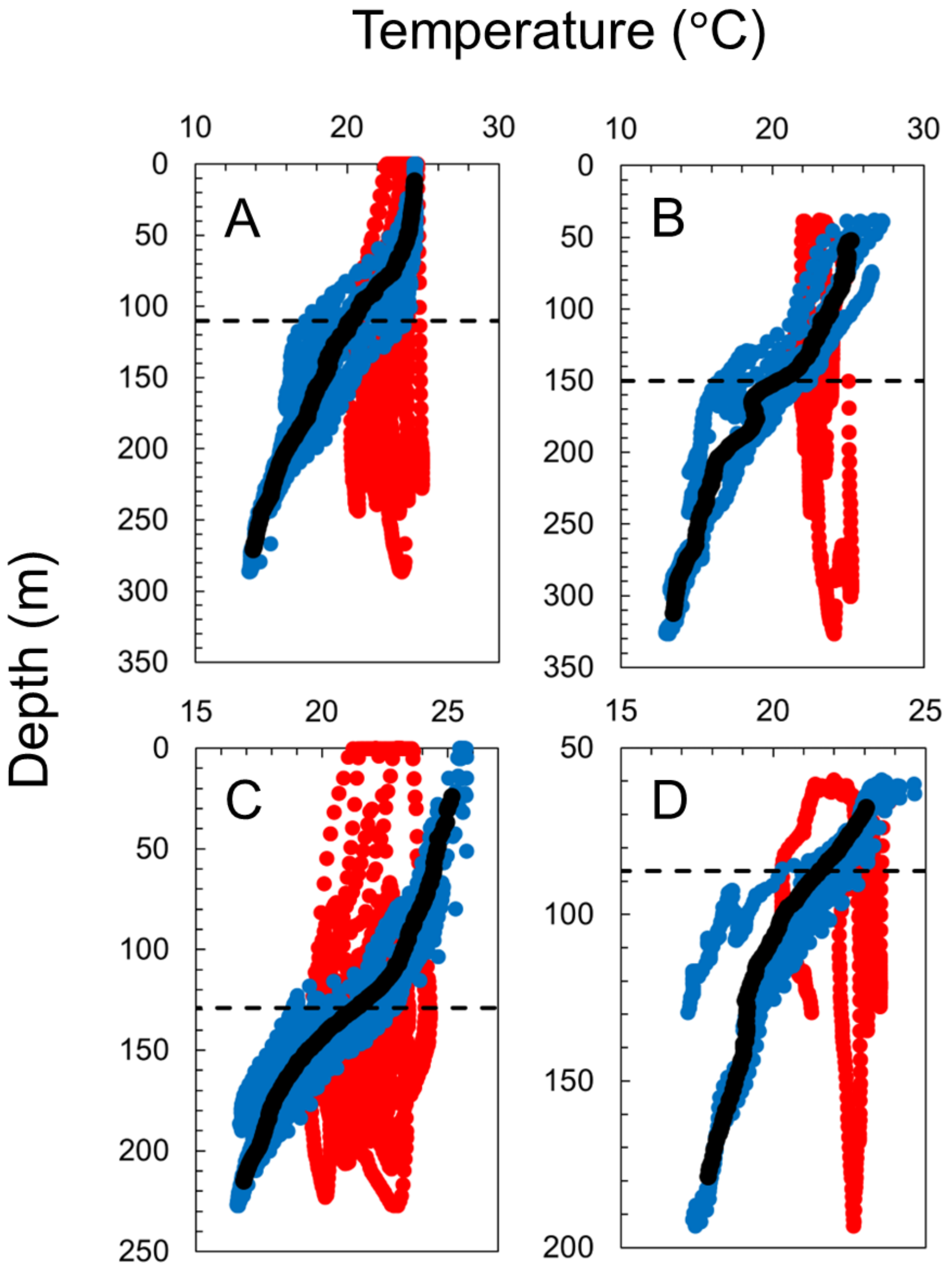

Figure S2. Depth-temperature profiles for Mako 1–4 (A–D). Muscle temperature (red), water temperature (blue), and smoothed water temperature (black) were plotted against depth. The dashed line represents the thermocline depth with the steepest temperature gradient.

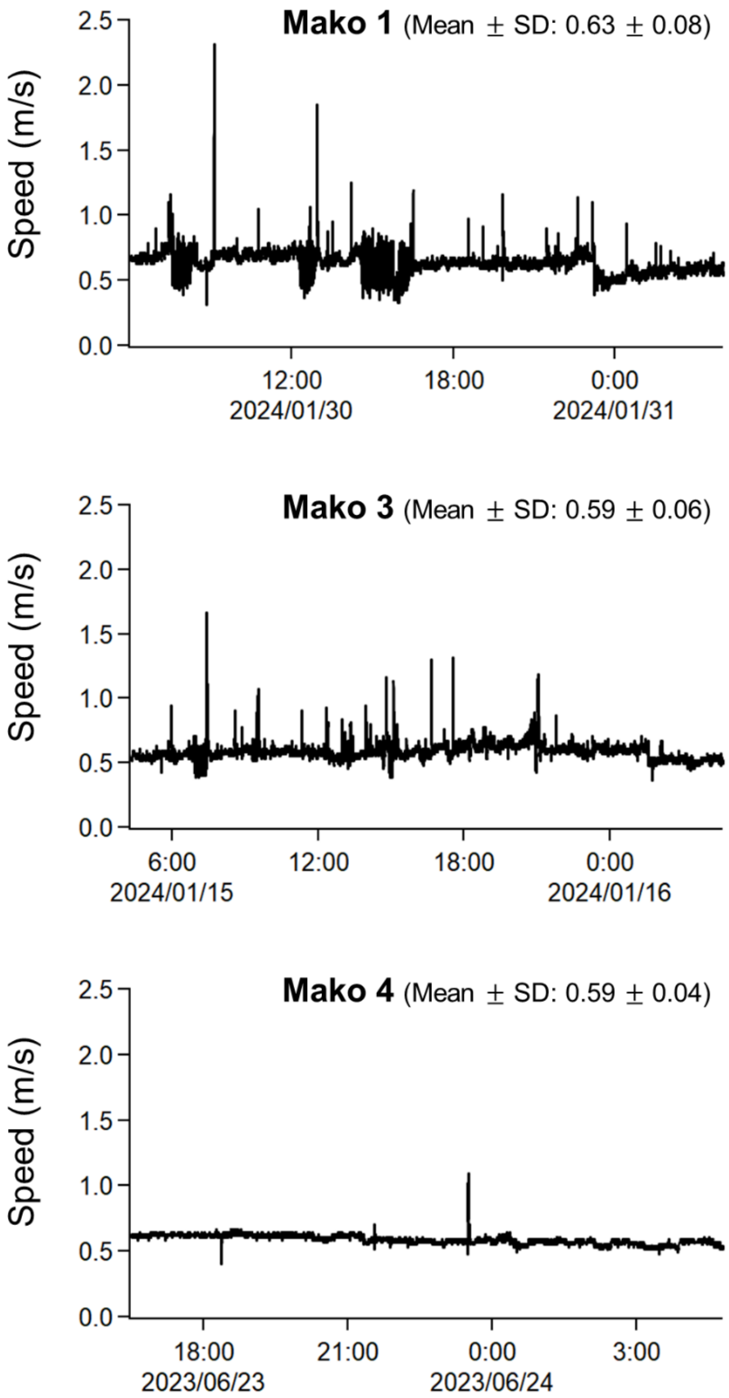

Figure S3. Swim speed of tagged mako sharks. Data for Mako 2 was lacking due to technical errors.

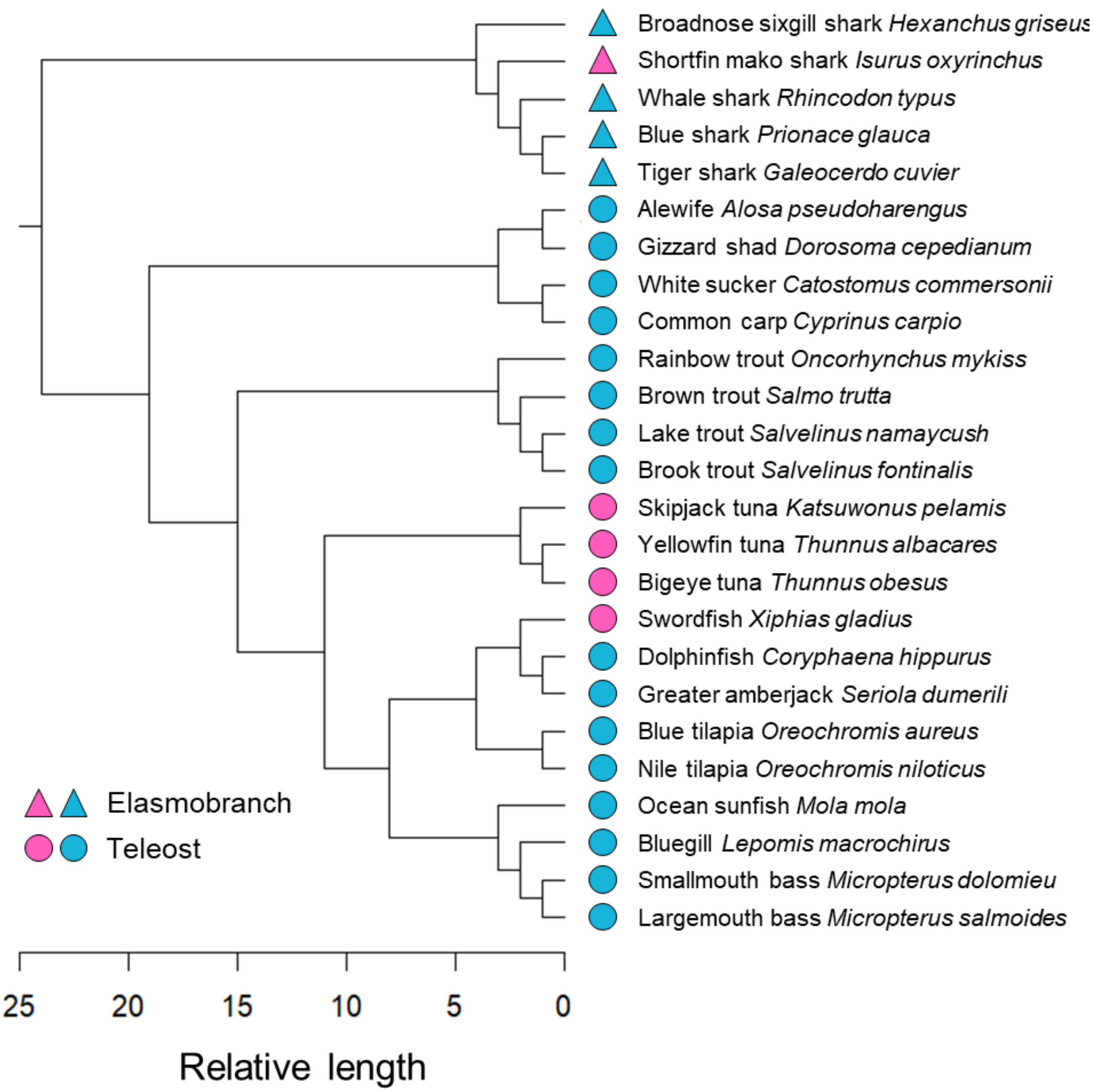

Figure S4. Phylogenetic tree used in comparative analyses. The pink and sky-blue markers represent regionally endothermic and ectothermic species, respectively.

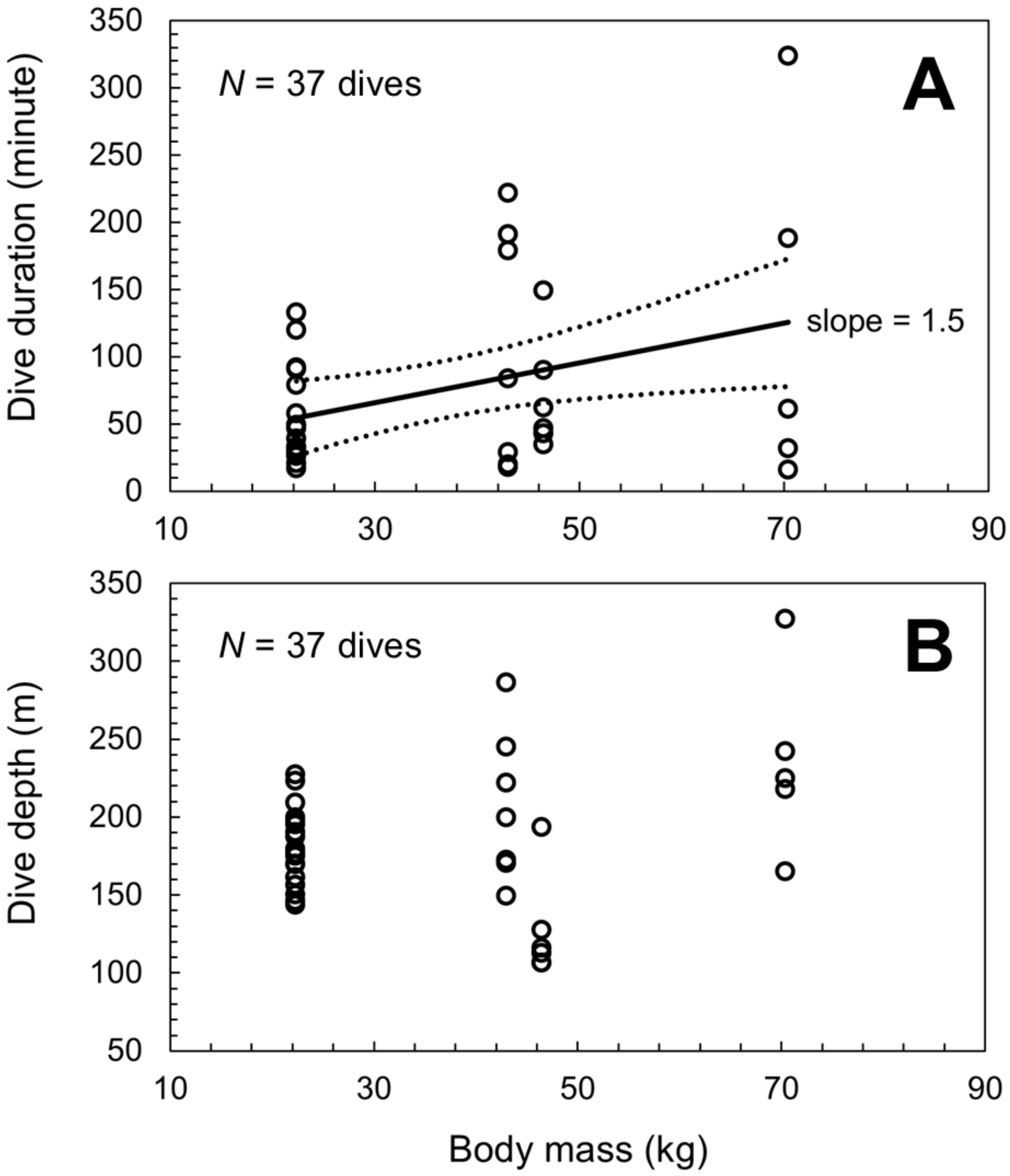

Figure S5. Body mass dependence of dive duration (A) and dive depth (B) in four tagged mako sharks. The dotted lines in A represent the upper and lower 95% confidence intervals. Dive duration was positively related with body mass (linear mixed model, 95%CI of the slope: 0.22–2.7), while dive depth was not (linear mixed model, 95%CI of the slope: -1.8–3.9).

Table S1. Summary of body mass and heat transfer coefficients for 25 fish species.

Species	n	Mass (kg)	k_{warm}	k_{cool}	k_{warm} / k_{cool}	Reference
Shortfin mako shark *Isurus oxyrinchus*	9	5.0–70.4	0.0056–0.17	0.0016–0.075	1.0–46.9	This study; Bernal, Sepulveda and Graham (2001)
Bigeye tuna *Thunnus obesus*	7	3.5–13.6	0.27–2.4	0.024–0.036	8.2–77.4	Holland and Sibert (1994); Malte *et al.* (2007)*
Yellowfin tuna *Thunnus albacares*	32	0.95–3.2	0.050–0.15	0.032–0.12	0.89–2.9	Dewar, Graham and Brill (1994); Aoki *et al.* (2020)
Skipjack tuna *Katsuwonus pelamis*	5	1.4–1.8	0.11–0.21	0.034–0.086	2.5–3.4	Lin *et al.* (2022)
Swordfish *Xiphias gladius*	8	32.0–140	0.025–0.10	0.0026–0.047	0.77–25.0	Stoehr *et al.* (2018)
Blue shark *Prionace glauca*	2	25.9–33.0	0.042–0.044	0.016–0.030	1.4–2.8	Watanabe, Nakamura and Chiang (2021)
Tiger shark *Galeocerdo cuvier*	1	35.0	0.034	0.017	2.0	This study
Whale shark *Rhincodon typus*	3	800–1600	0.0045–0.0081	0.0014–0.0034	2.2–3.3	Nakamura, Matsumoto and Sato (2020)
Bluntnose sixgill shark *Hexanchus griseus*	5	166–408	0.0053–0.0090	0.0053–0.0090	1.0	Coffey *et al.* (2020)
Dolphinfish *Coryphaena hippurus*	2	1.3–3.1	0.13–0.20	0.12–0.19	1.1	Furukawa *et al.* (2015)
Greater amberjack *Seriola dumerili*	7	7.0–16.9	0.052–0.088	0.037–0.063	1.3–1.7	Tone *et al.* (2022)
Blue tilapia *Oreochromis aureus*	2	0.041–0.14	0.25–0.54	0.27–0.56	0.93–0.96	Fechhelm and Neill (1982)
Nile tilapia *Oreochromis niloticus*	3	0.019–0.15	0.26–0.93	0.27–0.94	0.96–1.1	Fechhelm and Neill (1982)
Ocean sunfish *Mola mola*	6	28–360	0.0088–0.059	0.0022–0.040	1.5–6.5	Nakamura, Goto and Sato (2015); Nakamura and Yamada (2022)
Bluegill *Lepomis macrochirus*	5	0.022–0.058	0.44–0.83	0.44–0.74	1.0–1.1	Fechhelm and Neill (1982)
Largemouth bass *Micropterus salmoides*	8	0.52–2.7	0.076–0.19	0.054–0.16	1.1–1.6	Weller *et al.* (1984)
Smallmouth bass *Micropterus dolomieu*	1	0.32	0.17	0.16	1.1	Spigarelli, Thommes and Beitinger (1977)
Rainbow trout *Oncorhynchus mykiss*	1	0.31	0.14	0.11	1.3	Spigarelli, Thommes and Beitinger (1977)
Brook trout *Salvelinus fontinalis*	1	0.68	0.18	0.14	1.3	Spigarelli, Thommes and Beitinger (1977)

Lake trout *Salvelinus namaycush*	6	2.0–4.5	0.036–0.078	0.032–0.056	0.64–1.8	Spigarelli, Thommes and Beitinger (1977)
Brown trout *Salmo trutta*	24	0.20–5.9	0.033–0.38	0.020–0.27	0.76–2.7	Spigarelli, Thommes and Beitinger (1977)
Alewife *Alosa pseudoharengus*	25	0.010–0.050	0.54–2.9	0.37–2.4	0.79–2.0	Spigarelli, Thommes and Beitinger (1977)
Gizzard shad *Dorosoma cepedianum*	22	0.13	1.1	1.0	1.1	Beitinger, Thommes and Spigarelli (1977)†
Common carp *Cyprinus carpio*	3	2.2–4.3	0.075–0.099	0.027–0.059	1.3–3.7	Spigarelli, Thommes and Beitinger (1977)
White sucker *Catostomus commersonii*	1	0.57	0.15	0.24	0.63	Spigarelli, Thommes and Beitinger (1977)

*One individual presented in Holland and Sibert (1994) was excluded due to the limited recording duration of three hours with few warming phases.

†Only average values were available in the literature.

References

Aoki, Y., Aoki, A., Ohta, I. & Kitagawa, T. (2020) Physiological and behavioural thermoregulation of juvenile yellowfin tuna *Thunnus albacares* in subtropical waters. *Marine Biology,* **167,** 71. https://doi.org/10.1007/s00227-020-03679-w

Beitinger, T., Thommes, M. & Spigarelli, S. (1977) Relative roles of conduction and convection in the body temperature change of gizzard shad, *Dorosoma cepedianum*. *Comp. Biochem. Physiol., A;(United Kingdom),* **57**. https://doi.org/10.1016/0300-9629(77)90470-4

Bernal, D., Sepulveda, C. & Graham, J.B. (2001) Water-tunnel studies of heat balance in swimming mako sharks. *Journal of Experimental Biology,* **204,** 4043-4054. https://doi.org/10.1242/jeb.204.23.4043

Coffey, D.M., Royer, M.A., Meyer, C.G. & Holland, K.N. (2020) Diel patterns in swimming behavior of a vertically migrating deepwater shark, the bluntnose sixgill (*Hexanchus griseus*). *PLoS One,* **15,** e0228253. https://doi.org/10.1371/journal.pone.0228253

Dewar, H., Graham, J.B. & Brill, R.W. (1994) Studies of tropical tuna swimming performance in a large water tunnel: II. Thermoregulation. *Journal of Experimental Biology,* **192,** 33-44. https://doi.org/10.1242/jeb.192.1.33

Fechhelm, R.G. & Neill, W.H. (1982) Predicting body-core temperature in fish subjected to fluctuating ambient temperature. *Physiological Zoology,* **55,** 229-239.

Furukawa, S., Chiang, W., Watanabe, S., Hung, H., Lin, H., Yeh, H., Wang, S., Tone, K. & Kawabe, R. (2015) The first record of peritoneal cavity temperature recording in free-swimming dolphinfish *Coryphaena hippurus* by using archival tags, on the east coast of Taiwan. *J Aquacult Mar Biol,* **2,** 00032. http://dx.doi.org/10.15406/jamb.2015.02.00032

Holland, K.N. & Sibert, J.R. (1994) Physiological thermoregulation in bigeye tuna, *Thunnus obesus*. *Environmental Biology of Fishes,* **40,** 319-327. https://doi.org/10.1007/BF00002520

Lin, S.J., Chiang, W.C., Matsumoto, T., Chang, C.H., Ho, Y.S. & Ohta, F. (2022) Thermoregulation of skipjack tuna (*Katsuwonus pelamis*) using a heat budget model in eastern Taiwan. *Journal of Taiwan Fisheries Research,* **30**.

Malte, H., Larsen, C., Musyl, M. & Brill, R. (2007) Differential heating and cooling rates in bigeye tuna (*Thunnus obesus* Lowe): a model of non-steady state heat exchange. *Journal of Experimental Biology,* **210,** 2618-2626. https://doi.org/10.1242/jeb.003855

Nakamura, I., Goto, Y. & Sato, K. (2015) Ocean sunfish rewarm at the surface after deep excursions to forage for siphonophores. *Journal of Animal Ecology,* **84,** 590-603. https://doi.org/10.1111/1365-2656.12346

Nakamura, I., Matsumoto, R. & Sato, K. (2020) Body temperature stability in the whale shark, the world's largest fish. *Journal of Experimental Biology,* **223,** jeb210286. https://doi.org/10.1242/jeb.210286

Nakamura, I. & Yamada, M. (2022) Thermoregulation of ocean sunfish in a warmer sea suggests their ability to prevent heat loss in deep, cold foraging grounds. *Journal of Experimental Marine Biology and Ecology,* **546,** 151651. https://doi.org/10.1016/j.jembe.2021.151651

Spigarelli, S., Thommes, M. & Beitinger, T. (1977) The influence of body weight on heating and cooling of selected Lake Michigan fishes. *Comparative Biochemistry and Physiology Part A: Physiology,* **56,** 51-57. https://doi.org/10.1016/0300-9629(77)90441-8

Stoehr, A., St. Martin, J., Aalbers, S., Sepulveda, C. & Bernal, D. (2018) Free-swimming swordfish, *Xiphias gladius*, alter the rate of whole body heat transfer: morphological and physiological specializations for thermoregulation. *ICES Journal of Marine Science,* **75,** 858-870. https://doi.org/10.1093/icesjms/fsx163

Tone, K., Chiang, W.-C., Yeh, H.-M., Hsiao, S.-T., Li, C.-H., Komeyama, K., Kudo, K., Hasegawa, T., Sakamoto, T. & Nakamura, I. (2022) Two-way habitat use between reefs and open ocean in adult greater amberjack: evidence from biologging data. *Marine Ecology Progress Series,* **699,** 135-151. https://doi.org/10.3354/meps14169

Watanabe, Y.Y., Nakamura, I. & Chiang, W.C. (2021) Behavioural thermoregulation linked to foraging in blue sharks. *Marine Biology,* **168,** 1-10. https://doi.org/10.1007/s00227-021-03971-3

Weller, D.E., Anderson, D.J., DeAngelis, D.L. & Coutant, C.C. (1984) Rates of heat exchange in largemouth bass: experiment and model. *Physiological Zoology,* **57,** 413-427.
